# Executive function and quality of life in children and adolescents with type 1 diabetes: The mediating role of the Child Behavior Checklist Dysregulation Profile

**DOI:** 10.1007/s00431-025-06266-7

**Published:** 2025-06-25

**Authors:** Nuria Martin-Martinez, Irene Caro-Cañizares, Juan J. Carballo, Mara Parellada

**Affiliations:** 1 Department of Child and Adolescent Psychiatry, Institute of Psychiatry and Mental Health, Hospital General Universitario Gregorio Marañón, Instituto de Investigación Sanitaria Gregorio Marañón (IiSGM), CIBERSAM, ISCIII, School of Medicine, Universidad Complutense, Madrid, Spain; 2 Department of Psychology, School of Health and Educational Sciences, UDIMA (Universidad a Distancia de Madrid), 28400 Collado Villalba, Spain; 3 Department of Psychiatry, Fundación Jiménez Díaz Hospital, Madrid, Spain

**Keywords:** Type 1 diabetes, Executive function, Quality of life, Regulation, Adolescents

## Abstract

Type 1 diabetes (T1D) is a common childhood disease with a complex management that could adversely impact the quality of life of young sufferers. Executive functioning problems and psychopathology are factors that appear to have a detrimental effect on T1D-related quality of life (T1D-QoL). However, research on these factors is limited, and the pathways through which they do so remain unclear. This study aims to replicate the relationship between EF and T1D-QoL and to explore the possible mediating role of the clinical dysregulation profile (CBCL-DP) in this relationship. A total of 68 children and adolescents aged 10–18 living with T1D were recruited for this cross-sectional study. Parents reported on youth executive functioning, T1D-QoL, CBCL-DP, and socio-demographic data. In addition, clinical records of the youth were consulted to collect endocrinological information. Statistical analyses encompassed bivariate correlations, linear regressions, and bootstrap analyses to test the mediation model. Results demonstrate that executive function, CBCL-DP, and T1D-QoL are significantly correlated. The mediation model of the CBCL-DP variable in the relationship between executive function and T1D-QoL is significant. In linear regressions, executive function ceases to be significant on T1D-QoL when CBCL-DP is taken into account. The CBCL-DP significantly accounts for 55% of the variance in T1D-QoL. *Conclusion*: This study identifies the CBCL-DP as a full mediator between executive function and T1D-QoL, highlighting the importance of emotional and behavioral regulation for quality of life in youth with T1D. The CBCL-DP scale may be useful in identifying regulatory issues and guiding early interventions to improve outcomes in children and adolescents with T1D.

**Table:** 

**What is Known:** •* Previous studies have suggested that EF problems may negatively affect T1D-QoL; however, no studies have investigated the underlying mechanisms by which these variables are associated and the mediating role of CBCL-DP*.	
**What is New:** •*This study suggests that there is an absence of a direct association between EF problems and T1D-QoL and identifies the CBCL-DP as a complete mediator between these variables. Furthermore, the study indicates that clinical dysregulation acts as a risk factor for diminished T1D-QoL in children and adolescents with T1D*.

**What is Known:**

•* Previous studies have suggested that EF problems may negatively affect T1D-QoL; however, no studies have investigated the underlying mechanisms by which these variables are associated and the mediating role of CBCL-DP*.

**What is New:**

•*This study suggests that there is an absence of a direct association between EF problems and T1D-QoL and identifies the CBCL-DP as a complete mediator between these variables. Furthermore, the study indicates that clinical dysregulation acts as a risk factor for diminished T1D-QoL in children and adolescents with T1D*.

## Introduction

Type 1 diabetes (T1D) is one of the most complex and common chronic diseases in childhood, affecting around 0.05% of young people under 20 years worldwide [1]. T1D affects all aspects of an individual’s life, but especially the psychological sphere [2, 3], and has been associated with an increased risk of psychiatric comorbidities [4, 5].

The appropriate management of T1D, i.e., achieving and maintaining optimal HbA1c levels and treatment adherence over time, is important to reduce short and long-term health complications [6]. However, children and adolescents with diabetes face different management challenges that, combined with developmental challenges, can negatively impact their quality of life (QoL) [7–9]. Thus, a major goal of pediatric diabetes care is the achievement of an optimal balance that facilitates good daily T1D management without compromising the QoL of these young people [10, 11].

The natural transition of diabetes care from parent to youth occurs during adolescence and is accompanied by an increase in the cognitive demands necessary for T1D self-care [12]. Following the complex diabetes treatment regimen, which includes daily tasks such as checking and interpreting blood glucose levels and monitoring carbohydrate intake [13], involves higher-order cognitive skills such as executive function (EF). This set of skills facilitates planning and initiating action, organizing materials, shifting attention, and regulating impulses, thus playing an important role in the effective performance of T1D-related tasks [14]. In relation to QoL, to date, we have found only two studies investigating the impact of EF difficulties on QoL in youth with T1D. Both studies report that poor EF is related to worse QoL [12, 15].

Moreover, the presence of psychopathology among young people with T1D has also been associated with difficulties in diabetes management [16] and lower QoL [2, 17]. Compared to the non-diabetic population, young people with T1D are subject to an elevated risk of psychopathology, with emotional disorders and symptoms being of particular significance [2, 4, 18]. Thus, emotional management and regulation seem to play an important role in T1D. From a clinical perspective, severe emotional and behavioral dysregulation is defined as a clinical phenotype that does not fit neatly into any existing diagnostic category [19] and includes symptoms such as irritability, aggressiveness, hyperarousal, “affective storms,” and mood instability [20]. Recently, a profile has been developed to capture this severe mixed dysregulation phenotype from the general psychopathology assessment instrument known as the Child Behavior Checklist (CBCL) [21]. The negative impact of the so-called CBCL dysregulation profile (CBCL-DP) at the cognitive and psychosocial levels has previously been described in clinical juvenile populations and has been associated with greater EF difficulties and lower QoL [22, 23].

Although previous research has identified separately that both the presence of EF problems and psychopathology negatively affect QoL in children and adolescents with T1D, few studies have investigated these variables in combination and, to our knowledge, no previous work has studied the relationship between CBCL-DP and type 1 diabetes-related QoL (T1D-QoL). Thus, this work aims to contribute to the existing knowledge first by analyzing the relationship already found between EF and T1D-QoL, and second by testing whether CBCL-DP acts as a mediating factor in this relationship.

Based on the existing literature, we hypothesize that poorer EF is significantly associated with both lower total and domain-specific T1D-QoL. Similarly, given the independent relationships found between CBCL-DP and EF, as well as QoL in other juvenile samples, we expect to find the same separate results in a Spanish juvenile sample with T1D. Finally, we expect CBCL-DP to mediate the relationship between EF and total T1D-QoL.

## Materials and methods

### Subjects and procedures

This cross-sectional study included 68 children and adolescents with T1D, as well as their parents. Participants were recruited during their routine visits to the pediatric endocrinology consultation at the Gregorio Marañón and Fundación Jiménez Díaz University Hospitals (Madrid, Spain) between October 2020 and July 2023. Participants were included in the study if they were aged 10–18 years (both inclusive) and had been diagnosed with T1D for at least 3 years. Exclusion criteria included the inability of either the parents or children to comprehend the questionnaires administered, or any pathology that prevented participation in the study (e.g., severe developmental or cognitive disorders). The recruitment process involved a thorough review of youth clinical records by designated professionals. Those who did not present a clinical condition that could compromise the study and met the remaining inclusion criteria were invited to participate. All referred youth who agreed to participate were included in the study. Only one participant was excluded from the study for not completing the different parts of the evaluation.

Prior to participation in the study, written informed consent and assent for participation were obtained from parents (or legally authorized representatives) and youth, respectively. Data for the study were collected from the parents using the parent-report version of the tools used. The study was approved by the ethics committee of the Gregorio Marañón and Fundación Jiménez Díaz Hospitals.

### Measures

#### Socio-demographic and endocrinological information

Parents were interviewed to obtain their socio-demographic information, including participants’ sex, age, race, and parental education level (dichotomized as whether the parents had received higher education: Yes or No). Endocrinological information was collected from medical records of the youth (Table 1).

**Table 1:** Demographics, endocrinology, and clinical characteristics of the sample

	*n* (%)	*M* ± *SD*
Age		13.75 ± 2.35
Gender		
Female	37 (54.4)	
Male	31 (45.6)	
Race		
European	59 (86.8)	
Non-European	9 (13.2)	
Parental education (*n* = 65)		
1. Part grammar school	2 (3.1)	
2. Grammar school graduate	4 (6.2)	
3. Part high school	1 (1.5)	
4. High school graduate	17 (26.2)	
5. Part college or post high school training	10 (15.4)	
6. College graduate	9 (13.8)	
7. Post college	22 (33.8)	
Age at diabetes onset		7.24 ± 3.57
T1D duration		5.93 ± 2.96
HbA1c		7.94 ± 1.45
Treatment type		
Multiple daily injections	43 (63.2)	
Pump	25 (36.8)	
GEC (BRIEF-2)		53.44 ± 13.69
CBCL-DP		150.56 ± 30.78
T1D-QoL (PedsQL)		69.57 ± 17.37
Symptoms		71.02 ± 19.21
Treatment barriers		64.98 ± 25.24
Adherence		75.52 ± 21.47
Worry		66.79 ± 26.29
Communication		69.55 ± 17.37

*M* median, *SD* standard deviation, *GEC* Global Executive Composite, *CBCL-DP* Child Behavior Checklist Dysregulation Profile, *T1D-QoL* type 1 diabetes-related quality of life

#### T1D-related quality of life

The Spanish version of the PedsQL 3.0. [24] was used to assess each young person’s diabetes-specific health-related QoL according to their parent’s report. This instrument consists of five subscales (diabetes symptoms, treatment barriers, treatment adherence, worry, and communication), which are combined to form a total T1D-QoL score. The 28-item questionnaire assesses how much of a problem each item has been, utilizing a five-point Likert-type response scale ranging from 0 to 4 (0 = never; 1 = almost never; 2 = sometimes; 3 = often; 4 = almost always). The responses are then linearly transformed into an inverted scale ranging from 0 to 100 (0 = 100; 1 = 75; 2 = 50; 3 = 25; 4 = 0), with lower scores indicating greater diabetes-related problems and lower QoL associated with the disease. The score of each subscale is calculated through the mean of the transformed items comprising it (taking the requested data into account). The total scale and all subscales obtained good reliability (*α* = 0.92–0.74).

#### Executive function

The Spanish version of the Behavior Rating Inventory of Executive Function 2 (BRIEF-2) [25] is an ecological measure designed to examine youth EF problems in daily life. Each parent filled out a 63-item questionnaire on a three-point Likert scale (“never,” “sometimes,” or “frequently”) to indicate the frequency of each problem. The items were combined into a Global Executive Composite (GEC) score, with higher scores indicating worse EF. The T-scores from the manual were used for this study, with scores ≥ 70 considered clinically significant. In this study, the scale showed high internal consistency (*α* = 0.96).

#### Dysregulation profile

The CBCL dysregulation profile was used to assess this dysregulated phenotype. The CBCL is a widely used and standardized tool for the assessment of emotional and behavioral problems in young people. The CBCL (Spanish version) consists of 120 items rated by parents on a scale of 0 to 2 (0 = not true, 1 = sometimes true, 2 = very true or often true). The CBCL-DP has been previously developed and validated [26–30]. It is calculated from the sum of the T-scores of three subscales of the CBCL: anxiety/depression, attention problems, and aggression. Higher scores are associated with greater emotional and behavioral dysregulation problems. Consistent with previous literature [22, 31], dysregulation profiles were defined as T-scores ≥ 180 in this study. The CBCL-DP scale achieved a Cronbach’s *α* of 0.94, indicating good internal consistency.

### Statistical analysis

Descriptive analyses were evaluated, and bivariate associations between all study variables were performed through correlation analysis. Since the variables followed a nonparametric distribution, Spearman and point-biserial correlations were used to assess the strength of the relationship between the variables and to determine which variables to use as covariates. Linear regression analyses were conducted to examine the relationships between EF, CBCL-DP, and T1D-QoL. Socio-demographic variables, age at diabetes onset, parental educational level, and HbA1 were included as covariates in the initial analyses. The results showed that socio-demographic variables were no longer significant in the model, so they were excluded from the model to improve the overall model fit. Four separate linear models were developed to explore the relationship between the variables of interest including covariates (age at diabetes onset, HbA1c, and parental educational level) and a pairwise comparison of the resulting models was performed:

- Model 1: T1D-QoL − covariates.
- Model 2: T1D-QoL − covariates + GEC.
- Model 3: T1D-QoL − covariates + CBCL-DP.
- Model 4: T1D-QoL − covariates + GEC + CBCL-DP.

A mediation model was performed to assess the role of CBCL-DP in the relationship between EF and T1D-QoL. To test the mediation model, the standardized method of Baron and Kenny (1986) [32] was followed based on four criteria being met [33]: (1) the independent variable had to be correlated with the dependent variable; (2) the independent variable had to be correlated with the potential mediator; (3) the potential mediator had to be correlated with the dependent variable, controlling for the independent variable; and (4) once the three previous conditions were met, the correlation between the independent and the dependent variables had to decrease significantly with the inclusion of the potential mediator in the model.

Mediation analyses were conducted using bootstrap sampling methods. Bootstrapping is a nonparametric method for testing hypotheses, estimating effect size, and constructing confidence intervals without making any assumptions about the shape of the distribution (e.g., normality, which is necessary for classical parametric methods). It is obtained by taking a large number of samples with replacement of size *n* from the data (where *n* is the original sample size) [34], and it is regarded as being of great interest in psychology research [35]. In our study, 5000 iterations were applied in each calculation.

Once a mediation model is developed, a formal test is needed to determine the presence of the mediation effect [36]. Generally, the Sobel test is used; however, due to some limitations of the Sobel test, especially when applied to small samples [34], we also studied the indirect effects via bootstrapping.

In this work, a mediation model was developed in which T1D-QoL was taken as the dependent variable, GEC was taken as the independent variable, and CBCL-DP was taken as a possible mediating variable. In a second step, the mediation analysis was repeated including as covariates the sex and age of the youth, the educational level of the parents and the age of diabetes onset in the youth, due to their weight in the previous correlation and regression analyses. To perform the mediation analyses by bootstrapping, the INDIRECT macro [34] was installed in the SPSS program.

## Results

### Sample features

The final sample consisted of 68 participants (54.4% female) with a mean age of 13.75 ± 2.35 years. Most participants were European (86.8%). According to the Hollingshead scale (1975) [37], nearly half the families included at least one parent with higher education (see Table 1). Regarding T1D-related information, the mean age of onset was 7.24 ± 3.57 years of age and most participants were receiving multiple injection-based diabetes treatment. The sample had a mean of HbA1c of 7.94%, but only 16 subjects achieved values adequate to meet the glycemic control target recommended by the International Society for Pediatric and Adolescent Diabetes (ISPAD) in 2022 (HbA1c < 7) [38].

### Correlation and regression analyses

The correlations between the variables of interest are reported in Table 2. The GEC correlated positively and significantly with CBCL-DP (0.71; *p* < 0.01) and negatively with total T1D-QoL (− 0.42; *p* < 0.01) and the T1D-QoL subscales of symptoms, treatment barriers, and adherence. In addition, CBCL-DP correlated negatively and significantly with T1D-QoL (− 0.67; *p* < 0.01) and all subscales.

**Table 2:** Bivariate correlations between variables in the study

	1	2	3	4ª	5	6	7	8	9	10	11	12	13	14	15	16
1. Age	-															
2. Sex	0.05	-														
3. Race	0.21	− 0.08	-													
4. Parental educationª	0.08	0.04	0.20	-												
5. Age at diabetes onset	0.512**	0.11	0.11	0.15	-											
6. T1D duration	0.11	− 0.09	0.05	− 0.15	− 0.741**	-										
7. Treatment type	− 0.15	0.04	0.12	0.04	− 0.19	0.18	-									
8. HbA1c	0.370**	− 0.11	− 0.13	− 0.23	0.286*	− 0.06	− 0.453**	-								
9. GEC	0.05	0.01	− 0.13	0.05	0.21	− 0.12	− 0.241*	0.16	-							
10. CBCL-DP	− 0.04	0.04	− 0.18	− 0.08	0.12	− 0.08	− 0.03	0.10	0.713**	-						
11. T1D symptoms	− 0.12	0.13	0.23	0.297*	− 0.18	0.07	0.05	− 0.18	− 0.444**	− 0.664**	-					
12. Treatment barriers	− 0.284*	0.01	0.13	− 0.01	− 0.319**	0.10	0.18	− 0.21	− 0.462**	− 0.662**	0.570**	-				
13. Treatment adherence	− 0.16	0.14	0.24	0.19	− 0.14	− 0.01	0.07	− 0.18	− 0.417**	− 0.637**	0.520**	0.663**	-			
14. Worry	− 0.20	− 0.09	0.24	0.24	− 0.21	0.08	− 0.03	− 0.333**	− 0.061	− 0.247*	0.460**	0.260*	0.314**	-		
15. Communication	0.01	− 0.02	0.11	0.20	− 0.11	0.16	0.10	− 0.16	− 0.141	− 0.362**	0.442**	0.415**	0.356**	0.168	-	
16. Total T1D-QoL	− 0.20	0.06	0.249*	0.267*	− 0.285*	0.13	0.11	− 0.313**	− 0.424**	− 0.671**	0.805**	0.753**	0.721**	0.601**	0.699**	-

Spearman and point-biserial correlations were used as appropriate

Bold values indicate statistical significance (*p < 0.05, **p < 0.001)

*GEC* Global Executive Composite, *CBCL-DP* Child Behavior Checklist Dysregulation Profile, *T1D-QoL* type 1 diabetes-related quality of life

ª *n*= 65

Regarding the linear regression models, all four were significant: Model 1 accounted for 18% of the variance, Model 2 accounted for 29% of the variance, and Models 3 and 4 accounted for 55% of the variance. Furthermore, when introducing GEC and CBCL-DP into the models (together and separately), HbA1c was no longer significant in the models.

The pairwise comparison revealed the following:

- Comparing Model 1 (adjusted *R*^2^ = 0.177, *p* = 0.002) with Model 2 (adjusted *R*^2^ = 0.291, *p* < 0.001) showed that introducing GEC into the model contributes significantly to the model, while age of onset ceases to be a significant covariate in the model.
- Comparing Model 1 (adjusted *R*^2^ = 0.177, *p* = 0.002) with Model 3 (adjusted *R*^2^ = 0.551, *p* < 0.001) showed that CBCL-DP makes a significant contribution to the model, increasing the variance by 37%.
- Comparing Model 2 (adjusted *R*^2^ = 0.291, *p* < 0.001) with Model 4 (adjusted *R*^2^ = 0.551, *p* < 0.001) showed that taking GEC and CBCL-DP together, GEC ceases to be significant in the model, and CBCL-DP alone explains greater variance than GEC.
- Comparing Model 3 (adjusted *R*^2^ = 0.550, *p* < 0.001) with Model 4 (adjusted *R*^2^ = 0.550, *p* < 0.001) showed that taking GEC and CBCL-DP together does not make a greater contribution to the model with respect to the model that takes only CBCL-DP as an explanatory variable.

According to the results, the best-fitting model is Model 3, in which CBCL-DP and the covariates account for 55% of the variance of the total T1D-QoL.

### Mediation analysis

**Fig. 1:**
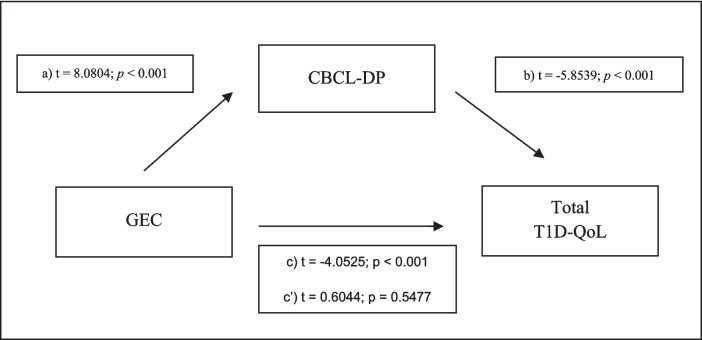
Dysregulation profile mediates the relationship between executive function and T1D-related quality of life. *N* = 68. (**a**) Correlation between the independent variable (GEC) and the proposed mediator (CBCL-DP); (**b**) effect of the proposed mediator (CBCL-DP) on the dependent variable (total T1D-QoL), controlling for the independent variable; (**c**) the total effect of the independent variable (GEC) on the dependent variable (total T1D-QoL), not controlling for the mediator; (c′) the effect of the independent variable (GEC) on the dependent variable (total T1D-QoL), controlling for the proposed mediator (CBCL-DP)

We followed standard methods to develop the mediation model, and our results meet the four criteria described above (see Fig. 1).

We found that (1) general executive function (GEC) correlates significantly with total quality of life (T1D-QoL); (2) GEC correlates significantly with the CBCL dysregulation profile; (3) the CBCL dysregulation profile correlates significantly with T1D-QoL, and the relationship remains significant when controlling for the effect of GEC; and (4) the relationship between GEC and T1D-QoL ceases to be significant when controlling for the potential mediator (CBCL-DP).

The bootstrap study of the indirect effect supports the mediation model, as the indirect effect is significantly different from zero at *p* < 0.05 (− 0.6636, CI − 0.9233 to − 0.4534). It is notable that when analyzing the inverse model, exchanging IV for the potential mediator, it does not hold as the 95% confidence interval of the indirect effect analysis contains zero (0.0284, [95% CI − 0.0857 to 0.1243]), supporting the specificity of the model presented in Fig. 1.

The second mediation model, which includes sex, age, parental educational level, and age of diabetes onset in youth as covariates, similarly meets the formal criteria for mediation analyses and the indirect effects study in turn supports the model (− 0.6790, 95% CI [− 0.9616 to − 0.4533]).

## Discussion

The aim of this study was to assess the relationship between executive function and diabetes-related quality of life, making it the first study to explore the possible mediating role of the emotional and behavioral dysregulation profile in a Spanish sample of children and adolescents with T1D.

As predicted, the initial analyses supported our hypothesis by showing that EF and T1D-QoL are significantly associated. However, when introducing the dysregulated phenotype as a mediator, this correlation disappears. Mediation analyses revealed that CBCL-DP acts as a full mediator of the relationship between EF and total T1D-QoL. This finding contributes to the extant knowledge regarding the relationship between EF and T1D-QoL to date, suggesting that these two factors are not directly related. Our results support the idea that adolescents with T1D who exhibit greater difficulties in EF are more likely to display a more dysregulated clinical profile that involves behaviors that may hinder treatment compliance.

Examining the relationships between EF and T1D-QoL without considering the influence of CBCL-DP, the results show that greater EF difficulties are associated with poorer total T1D-QoL; these findings are consistent with those reported in studies involving juvenile samples with T1D [12, 15]. However, related to T1D-QoL subscales, EF is only significantly associated with the symptoms, treatment barriers, and adherence subscales. It is possible that the influence of EF varies across subscales depending on the content they assess. Our findings reveal a stronger correlation between the EF and T1D-QoL subscales that are more reliant on regulated and organized compliance with treatment. It is noteworthy that these same subscales demonstrate the strongest association with CBCL-DP. Therefore, given the mediating role of CBCL-DP, it is reasonable to hypothesize that these relationships follow the same pathway of association as that found with total T1D-QoL. To the best of our knowledge, these relationships have not been investigated before, so further studies are needed. On the other hand, with regard to T1D-QoL, it is noteworthy that, in contrast to the extant literature, no associations were identified between treatment modality and T1D-QoL. According to Blair et al. (2018), this could be explained by the level of satisfaction among youth with their T1D treatment, whether using a pump or multiple daily injections. Further studies are required to establish a more comprehensive understanding of the relationship between these two variables [39].

Concerning the associations identified between CBCL-DP and EF, as well as T1D-QoL, the results support our hypothesis, consistent with those reported in other non-diabetic clinical youth samples [22, 23]. The results show that a more dysregulated phenotype is associated with poorer EF, in addition to worse outcomes in all aspects of QoL in people with T1D, acting as a risk factor for a more problematic perception of the disease. This finding is particularly relevant since CBCL-DP seems to have an important explanatory role in T1D-QoL. The lack of a relationship between EF and T1D-QoL seems to strengthen once CBCL-DP is taken into account in the secondary regression analyses. This may indicate that a healthier and less dysfunctional experience of T1D may be more closely associated with the emotional management strategies employed by young people than with more cognitive aspects. Within the framework of hot and cool executive functions [40], these emotional and behavioral management strategies could be more closely aligned with hot EF processes, such as affective regulation and decision-making in emotionally salient contexts, than with cool, purely cognitive EF component.

Improvement of QoL is one of the main goals of any intervention. Given the growing scientific evidence on the negative consequences of both the presence of psychopathology [2, 17] and difficulties in EF among young people with T1D [12, 15, 41–43], it is strongly recommended that regular assessment of these types of aspects be included during endocrine consultations using validated instruments. According to the associations found in this study, clinical emotional dysregulation could be a relevant risk factor for QoL in diabetes. This finding requires replication, but if this is gained, emphasis on helping children to regulate their emotions and behavior could become a principal objective of intervention. Identification of other variables that influence the QoL of these children may therefore be useful for intervention plans. The full mediation found in this work suggests two possible avenues for intervention to improve QoL in diabetes. The first would involve neurocognitive work adapted to daily life to train EF. Thus, according to our results, an improvement in EF would be accompanied by better clinical regulation and thus better QoL in diabetes. The second type of intervention integrates psychotherapeutic work on the emotional, behavioral, and cognitive aspects related to clinical regulation, in which the participation of the family could also be considered.

Despite the findings of this study, several limitations must be considered when interpreting the results. Firstly, the cross-sectional design does not permit statements about causality between the variables. Further research using prospective longitudinal designs is needed to clarify the direction of the associations, as well as to test for the possibility that intervening in one factor (i.e., DP or EF) improves the desired outcome (T1D-QoL). On the other hand, the sample size is small, which limits our ability to detect small effects, so the results should be interpreted with some caution. Future studies with larger samples may be useful as such studies could permit the use of more complex models and analyses that include more variables, such as those related to family, since several studies have confirmed the importance of parents in diabetes outcomes [44–48] and the QoL of these youth [49]. Additionally, treatment adherence was assessed using a subscale of the PedsQL T1D Module, without the use of specific instruments designed to evaluate this variable in greater detail. Future studies could benefit from incorporating dedicated adherence measures to achieve a more comprehensive assessment. With respect to the assessment of comorbidities and complications, it was not adequately performed. Consequently, this measure was excluded from the analyses. Careful data collection is essential in future research to avoid potential confounding effects. It is also important to mention that we chose to use parent-reported measures to assess the main study variables. Although self-reports may be the best representation of how youth experience T1D in terms of QoL, parental perceptions are of interest as they may influence healthcare utilization [50]. With regard to EF in the juvenile T1D population, prior studies using questionnaires have been mainly conducted through parental reporting [14]. Furthermore, the questionnaire used (BRIEF-2) is a well-established tool for assessing problems with EF in daily life [51]. Unlike assessments using neuropsychological tests that provide information on “pure” cognitive abilities, this type of questionnaire describes the application of these abilities in daily life [52, 53]—information that is of particular interest in the diabetic population as a way of determining success in achieving therapeutic goals (e.g., adherence and glycemic control) depending on the use of EF. In future research, the use of diabetes-specific instruments such as the Diabetes Related Executive Functioning Scale questionnaire may be more useful and accurate [43]; however, this questionnaire has not been adapted for all languages.

## Conclusion

To our knowledge, this is the first study to examine CBCL-DP as a total mediator between EF and T1D-QoF in youth with T1D. It hereby highlights that the capacity of young people to regulate their emotions and behaviors plays a crucial role in their T1D-QoL. In addition, our results also suggest that EF appears to influence T1D-QoL only through CBCL-DP.

Consequently, the CBCL-DP scale may be a useful, easy-to-use instrument in pediatric endocrine consultations to identify regulatory issues that hinder optimal disease-related QoL. Early detection of this phenotype may facilitate the implementation of suitable interventions that enhance the prognosis of children and adolescents with T1D.

## Data Availability

All data are available upon reasonable request emailed to the corresponding author.

## Abbreviations

BRIEF-2: Behavior Rating Inventory of Executive Function 2
CBCL: Child Behavior Checklist
CBCL-DP: Child Behavior Checklist Dysregulation Profile
EF: Executive function
GEC: Global Executive Composite
QoL: Quality of life
T1D: Type 1 diabetes
T1D-QoL: Type 1 diabetes-related QoL
